# Phytochemical Content and Antioxidant Activity of *Malus domestica* Borkh Peel Extracts

**DOI:** 10.3390/molecules26247636

**Published:** 2021-12-16

**Authors:** Melnic Vasile, Andrea Bunea, Chira Romeo Ioan, Bunea Claudiu Ioan, Sonia Socaci, Mitre Viorel

**Affiliations:** 1 Faculty of Horticulture, Department of Horticulture and Landscaping, University of Agricultural Sciences and Veterinary Medicine, 3-5 Mănăstur Street, 400372 Cluj-Napoca, Romania; vasilemelnic91@gmail.com (M.V.); claudiu.bunea@usamvcluj.ro (B.C.I.); viorel.mitre@usamvcluj.ro (M.V.); 2 Faculty of Animal Science and Biotechnologies, Department of Fundamental Sciences, University of Agricultural Sciences and Veterinary Medicine, 400372 Cluj-Napoca, Romania; 3 University of Medicine and Pharmacy “Iuliu Hatieganu”, 3–5 Clinicilor Street, 400006 Cluj-Napoca, Romania; romeochira@yahoo.com; 4 Faculty of Food Science and Technology, Department of Food Science, University of Agricultural Sciences and Veterinary Medicine, 400372 Cluj-Napoca, Romania; sonia.socaci@usamvcluj.ro

**Keywords:** apple peel, carotenoids, phenolic compounds, anthocyanins, antioxidant activity, FRAP, ABTS

## Abstract

Apple is an important dietary source of carotenoids and phenolic compounds, and its regular consumption is associated with several health benefits. The aim of this study was to evaluate the phytochemical composition of fresh peels of four red-skinned (“Champion”, “Generos”, “Idared”, “Florina”) and two yellow-skinned (“Golden Delicious”, “Reinette Simirenko”) apple varieties. Antioxidant activity of apple peel extracts was determined by ferric reducing antioxidant power (FRAP) and ABTS radical scavenging capacity assays. Total carotenoid and polyphenolic contents were determined spectrophotometrically, while the profile of individual carotenoids and anthocyanins (in red-skinned varieties) was analyzed using high-performance liquid chromatography coupled to a photodiode array detector (HPLC-PDA). Carotenoid composition was specific for each variety, and total carotenoid content was slightly higher in yellow-skinned apple peels compared to red-skinned varieties. In contrast, total phenolic content was higher in the peels of red-skinned cultivars. Anthocyanin profile was predominated by cyanidin-3-*O*-galactoside. Antioxidant potential followed the trend of the total polyphenolic content, being highest in “Florina”, as measured by both FRAP and ABTS assays. Our results demonstrated apple peels have high phytochemical content with diverse compositions, and their regular consumption can be an excellent source of antioxidants.

## 1. Introduction

The domesticated apple (*Malus domestica* Borkh) is one of the most popular and widely cultivated fruits worldwide. It is an important dietary source of phenolic compounds with strong antioxidant activity compared to other fruits, and its consumption has been linked with improved health benefits and reduced risk of degenerative diseases [1,2]. This biological impact of apples, similar to that of many other fruits, may be related largely to the presence of antioxidants.

Polyphenols closely affect the quality characteristics of fresh fruits and their processed products. The concentration of individual phenolic compounds in apples is variable and depends on the cultivar, fruit maturity, cultivation method, soil and climatic conditions, and other factors [2]. Thus, data obtained in original studies performed on different cultivars under different conditions cannot be directly compared. Storage time and other postharvest conditions also affect the level of phenolic compounds and associated antioxidant capacity of the edible fruits and fruit products [3,4]. High levels of phenolic compounds in fruits are associated with elevated antioxidant activity [5]. Common phenolic compounds with strong antioxidant potential identified in apples are procyanidins, chlorogenic acids, flavonoids, hydroxycinnamic acids, anthocyanins, and quercetins, and these compounds are mainly concentrated in the skin [6,7]. Therefore, consumption of apple with peel is highly recommended, as it confers various in vitro bioactivities, higher than the flesh of the fruit [1,8]. For instance, polyphenol-rich apple peel extracts demonstrated antihypertensive, antidiabetic, anti-inflammatory, and antiproliferative properties [6,9,10] and effectively attenuated arsenic trioxide induced cardiotoxicity in H9c2 cells [11]. Moreover, phenolic compounds can have therapeutic benefits against several human diseases, such as cancer, obesity, diabetes, asthma, or cardiovascular diseases [12,13,14].

Red-skinned apples are receiving increased attention because of their high anthocyanin contents, which are also concentrated in the peel [15]. Although the pigments associated with color can vary, compositions of anthocyanins within the phenolic classification are considered the major determinants of apple skin reddening [16]. The anthocyanin content and color development of apples are continuously increasing during fruit maturation and are influenced by light, temperature, nutrition, and genetic factors [17,18]. The genetic mechanisms behind anthocyanin synthesis are complex, highly specific, and the subject of continuous research [15]. Anthocyanin formation was positively associated with phenylalanine ammonia-lyase and UDPG-Galactose flavonoid-3-*O*-glycosyltransferase activity [19,20]. Although their synthesis is strongly light dependent, anthocyanins are also involved in the defense against fruit damage, likely via protecting chlorophylls from photodestruction [21].

In apples, carotenoid concentration is low compared to other commercial fruits; however, the chemistry and bioavailability of carotenoids are extensively studied due to their nutritional and medicinal value [22,23]. Carotenoids have important physiological functions, such as provitamin A activity, antibacterial and antioxidant activity, and enhancement of immune system function by acting as immunomodulators [24,25,26]. They have protective effects against diabetes mellitus, cancer, and cardiovascular diseases [27]. The concentration of carotenoids in apple components is cultivar specific [28,29] and is greatly influenced by several factors, such as light exposure [30], harvesting conditions [31], or storing and processing conditions [32,33], but it can also be enhanced by genetic engineering [34,35].

**Figure 1:**
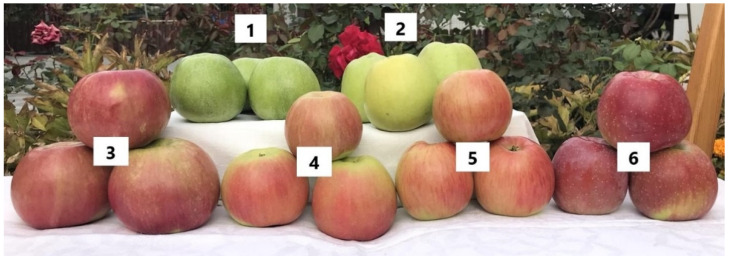
Apple cultivars analyzed in this study: 1—Reinette Simirenko; 2—Golden Delicious; 3—Idared; 4—Generos; 5—Champion; 6—Florina.

The aim of this study was to characterize the phytochemical composition of the peels of six different apple cultivars of *Malus domestica* Borkh grown in the Central region of Moldova. The individual carotenoid and anthocyanin composition of red-skinned “Champion”, “Generos”, “Florina”, and “Idared” varieties and yellow-skinned “Golden Delicious” and “Reinette Simirenko” varieties (Figure 1) were directly investigated by high-performance liquid chromatography coupled to a photodiode array detector (HPLC-PDA). Spectrophotometric methods were used to evaluate the total polyphenol content (TPC) and antioxidant potential of peel extracts by ferric reducing antioxidant power (FRAP) and 2,2′-azino-bis (3-ethylbenzothiazoline-6-sulfonic acid) (ABTS) radical scavenging activity assays.

## 2. Results and Discussion

### 2.1. Carotenoid Content of Apple Peels

**Figure 2:**
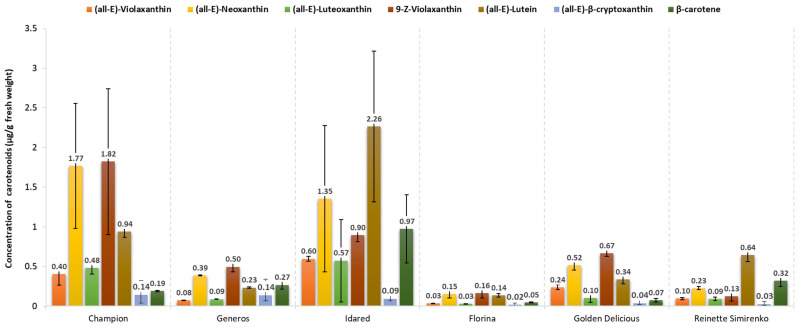
Concentration of individual carotenoids in red- and yellow-skinned apple varieties. Error bars depict standard deviation.

Carotenoids participate in light harvesting and are recognized as powerful antioxidants [36]. In previous studies, total carotenoid content has been reported to be higher in peels compared with flesh [1,8,28,37], indicating the potentially higher bioactivity of the former. All the cultivars in our study were characterized by similar pigment profiles, composed of neoxanthin (all-*E*), violaxanthin (all-*E*, 9-*Z*), lutein, (all-*E*), luteoxanthin (all-*E*), β-cryptoxanthin (all-*E*), and β-carotene, though the concentration of individual carotenoids differed between apple peel varieties (Figure 2). Published chromatographic data of carotenoids were used to identify and quantify each carotenoid in the samples. Neoxanthin and (9-*Z*)-violaxanthin were among the most dominant carotenoids in all red-skinned varieties. The highest values were detected in “Champion” (1.77 µg/g fresh weight [FW] and 1.82 µg/g FW, respectively) and the lowest in “Florina” (0.15 µg/g FW and 0.16 µg/g FW, respectively) (Figure 2). “Idared” was the variety with the highest lutein and β-carotene concentrations (2.26 µg/g FW and 0.97 µg/g FW). Among yellow-skinned varieties, 9-Z-violaxanthin had the highest concentration (0.67 µg/g FW) in “Golden delicious”, followed by neoxanthin (0.52 µg/g FW) and lutein (0.34 µg/g FW). In the “Reinette Simirenko” cultivar, the highest concentration was detected for lutein (0.64 µg/g FW), followed by β-carotene (0.32 µg/g FW). A similar carotenoid profile has been reported for other red-, yellow-, and green-skinned apple varieties, with lutein, violaxanthin, neoxanthin, and β-carotene being the main free carotenoids [28]. Merzlyak et al. reported a high carotenoid content for the “Reinette Simirenko” peel (2.38 nmol/cm^2^), the highest among green-skinned varieties analyzed in the study (“Golden delicious” and “Antonovka”) [38]. The same group also reported a decreased carotenoid content in the sunburned area of “Reinette Simirenko” compared to the unexposed area [39]. The carotenoid profile of apple peels undergoes considerable changes during ripening, though available literature is controversial on this matter. Some reports have shown that elevated sunlight induces carotenoid synthesis in apple peels during on-tree ripening, which continues postharvest and is more pronounced in the sunburned area than in the partially sunburned or nonsunburned areas [30,40,41]. Other researchers reported no correlation between carotenoid content and fruit position on the apple canopy [42] or reported even a continuous decrease in carotenoid concentration [43]. The application of urea also increased the carotenoid concentration in apple skin [17].

### 2.2. Total Phenolics Content of Apple Peels

TPC ranged from 2056 to 2723 mg gallic acid equivalent (GAE)/kg FW among red-skinned varieties and was higher than those obtained from yellow-skinned apples (Table 1). The highest value was found in red-fleshed “Florina” and the lowest value in yellow-skinned “Reinette Simirenko”. These TPC values are comparable with those reported for red-skinned, red-fleshed “Xinjiang” varieties (2062–2815 mg GAE/kg FW) [44]. The same study showed a significantly higher TPC concentration (2899 mg GAE/kg FW) for “Golden Delicious” apples skin extracts than our analysis (1600 mg GAE/kg FW), which was slightly higher than red-skinned varieties [44]. Other studies that used HPLC methods for the quantification of total phenolics found a TPC of 30.0–140.7 mg/100 g FW for “Idared” and 31.8–117.0 mg/100 g FW for “Champion” cultivated in Poland [4,45], 5 mg GAE/g dry weight for “Idared” and 12 mg GAE/g dry weight for “Golden Delicious” cultivated in Croatia [46], 1204–1374 mg/kg FW for “Golden Delicious” cultivated in Italy [47], and 1265 µg GAE/g FW for “Golden Delicious” and 1479 µg GAE/g FW for “Idared” cultivated in Canada [7]. Studies have shown that many heritage cultivars have higher phenolic levels than new commercial cultivars [5,46]. In the study conducted by Duda-Chodak et al., the polyphenol content of “Idared” and “Champion” peels decreased during ripening from 84.7 to 30.0 mg/100 g FW and from 69.0 to 31.8 mg/100 g FW, respectively, suggesting that unripe apple are more valuable for polyphenol extraction. Subsequently, polyphenolic content increased to 38.4 mg/100 g FW (“Idared”) and 46.8 mg/100 g FW (“Champion”) during long-term storage (112 days) in cold stores. This indicates that long-term-stored apples are still precious materials [4].

A clear influence of fruit position in the tree canopy on the TPC was demonstrated: TPC values for “Fuji” apple peels were lower for fruits from the inner part of the canopy (1685 mg GAE/kg FW) than for outer fruit (2264 mg GAE/kg FW) and top fruit (2767 mg GAE/kg FW) [42]. TPC also depends on different processing factors, such as the drying system and extraction solvent used. The optimal processing conditions of apple peels to preserve the phenolic compounds were proposed to be blanching and freeze-drying. The loss in TPC was reported during oven-drying compared with the TPC of air-dried and freeze-dried peels [37,48]. Additionally, TPC decreased significantly following postharvest UV-B treatment [49].

#### Anthocyanin Content of Apple Peels

The concentration of anthocyanins was determined for red-skinned apple varieties (Table 1) using HPLC-PDA. Three types of glycosylated cyanidins, cyanidin-3-*O*-galactoside, cyanidin-3-*O*-glucoside, and cyanidin-3-*O*-arabinoside, were detected. Cyanidin-3-*O*-galactoside was the predominant anthocyanin in all four varieties, covering >83% of the total anthocyanins. This is in line with results from previous studies conducted on both traditional and new varieties [44,50,51,52]. Among varieties, the highest cyanidin-3-*O*-galactoside and cyanidin-3-*O*-glucoside concentrations were identified in the “Florina” cultivar, significantly higher than in other species. “Idared” and “Champion” varieties contained similar amounts of cyanidin-3-*O*-galactoside. “Idared” had the highest cyanidin-3-*O*-arabinoside content, while in the “Champion” variety, only traces of cyanidin-3-*O*-arabinoside were detected. In a recent study conducted in Slovenia, postharvest accumulation of cyanidin-3-*O*-galactoside and cyanidin-3-*O*-arabinoside in “Idared” was successfully induced by irradiation with blue light, resulting in significantly higher concentrations than what we observed herein [51]. In another study from the United States, the anthocyanin content of “Idared” apple peels was reported to be significantly higher than the content of other apple peels [8].

Accumulation of anthocyanins occurs at two stages during the growth of the fruit: in young fruitlets, during cell division, and in fully developed apples, during maturation. Several chemical substances (e.g., ethephon, an ethylene releasing agent) are used to accelerate red color formation, while urea, ABG-3168, and gibberellic acid delay the formation of anthocyanins [17,53]. Precooling of apple, surface-coating with neem oil, and shrink-wrapped tray packing were effective to preserve the anthocyanin content of the fruit and allowed long-term storage up to 150 days [54]. Additionally, the anthocyanin content of blanched and freeze-dried “Rome Beauty” apple peels has been shown to be approximately 14-fold higher than the anthocyanin content of the fresh peels [37].

### 2.3. Antioxidant Activity

**Figure 3:**
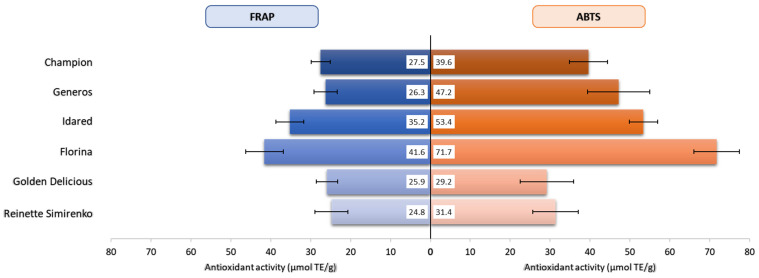
Antioxidant activity as measured by ferric reducing antioxidant power (FRAP) and scavenging effect on ABTS radical. TE, Trolox equivalent. Error bars depict standard deviations.

Several methods have been established for the evaluation of antioxidant activity of apples. As these methods differ in their reaction characteristics and mechanism, there is no universal assay that can accurately describe the antioxidant potential of all compounds in a complex system. Here, the FRAP and ABTS assays were used to measure the antioxidant activity of red- and yellow-skinned apple varieties. Values ranged from 24.8 µmol Trolox equivalent (TE)/g (“Reinette Simirenko”) to 41.6 µmol TE/g (“Florina”) as measured by FRAP and from 29.2 µmol TE/g (“Golden Delicious”) to 71.7 µmol TE/g (“Florina”) as measured by ABTS assay (Figure 3).

In accordance with phenolic composition, antioxidant activity varies considerably between the part of the fruit and is significantly higher in the peel than in the flesh or the whole fruit [8,55]. The strong correlation between the phenolic content of apples and their antioxidant activity is well established [56], and it is also supported by the present study. The “Florina” cultivar with the highest TPC also presented the highest antioxidant potential in both FRAP and ABTS assays, followed by Idared (35.2 and 53.4 µmol TE/g) (Figure 3). Although yellow-skinned varieties “Golden Delicious” and “Reinette Simirenko” had the lowest TPC, their redox potentials in the FRAP assay were comparable with the activity measured for red-skinned “Champion” and “Generos” varieties, which further supports the redox capacity of carotenoids. In the ABTS assay, a more pronounced contrast of scavenging activity was detected between red- and yellow-skinned varieties, with radical scavenging potential being higher in the former. This might be related to the methodical differences between assays.

Other studies that used the same assays reported similar antioxidant profiles for the peels of local apple species from China [44] or Turkey [5], while slightly lower values were reported for new species from Brazil [55]. A high level of TPC among local and commercial cultivars, including “Golden Delicious”, “Idared”, and “Champion”, analyzed from Poland, has shown a positive correlation with ABTS scavenging activity, slightly lower TPC in “Idared” and “Champion”, which resulted in significantly lower ABTS activity compared to local cultivars [45]. In parallel with the TPC decrease, the capacity to scavenge ABTS radicals also decreased in the peels of “Idared” and “Champion” from 693 to 306 mg TE/100 g FW during fruit ripening, but was subsequently increased to 424 (“Idared”) and 621 mg TE/100 g FW (“Champion”) after 112 days of cold storage [4]. Another study conducted in India found a positive correlation between apple cultivars growing in different altitudes, their phenolic content, and, consequently, their FRAP and ABTS activities [57].

**Figure 4:**
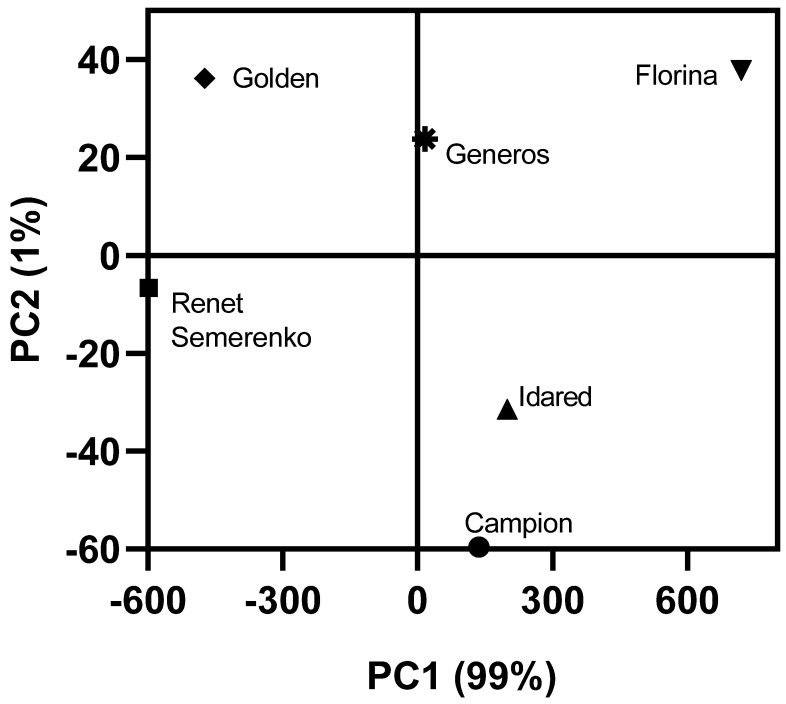
Principal components analysis biplots of apple cultivars based on their carotenoids and anthocyanins content, antioxidant activity. The first two components together explained 99% of the data variation.

Multivariate analysis, such as principal component analysis (PCA), is one of the most used methods for the analysis of complex data sets, e.g., the chemical composition of samples. In this study, PCA was carried out in order to assess the interrelationships between the apple cultivars, highlighting their similarities and differences. Thus, by applying this unsupervised method of pattern recognition on all data, the two principal components explained 100% of the overall variance (99% and 1% for PC1 and PC2, respectively) dividing the analyzed cultivars into distinct clusters (Figure 4). From the correlation loadings, the factors that most contributed to apple cultivar discrimination were their overall antioxidant capacity, anthocyanin profile, and luteoxanthin and lutein content.

## 3. Materials and Methods

### 3.1. Materials

All chemicals and reagents were of analytical grade, and the ultrapure water (18 MΩ cm resistance) used was treated in a Milli-Q water purification system. Carotenoid standards β-carotene, lutein, and zeaxanthin (purity ≥98%, ≥95%, and ≥98%, respectively) were acquired from Extrasynthese.

Apple cultivars grown in Jora de Mijloc, Orhei District, Moldova, were analyzed in this study (Figure 1). The apples were harvested at commercial maturity. The orchard was established in 2009. The planting distance between the rows is 4 m and 1 m between the trees in a row, which corresponds to a density of 2500 trees/ha. For each cultivar, 4 randomly chosen trees were selected in 4 replicates (n = 16) with similar fruit loads. The apples (100 apples/cultivar) were harvested at commercial maturity, 150 days after pollination. The evaluation of fruit maturity was based on skin color determination, fruit firmness, and soluble solid content (sugar content), which were determined by using a handheld fruit penetrometer and refractometer (data not shown). Fruits were loosely packed inside conventional modular bulk containers with polyliners and stored at 0 °C, 80–90% relative humidity. The humidity inside the polyliner was approximately 95%. The air was exchanged with fans four times daily to remove ethylene. All subsequent analysis was performed after the apples were washed under tap water, and the peels were removed mechanically with a hand peeler.

### 3.2. Extraction of Carotenoids

Carotenoids were extracted from apple peels (5 g) using the procedure described by Schlatterer and Breithaupt [58]. The peels were homogenized and extracted three times with a mixture of methanol/ethyl acetate/petroleum ether (1:1:1, *v*/*v*/*v*). The combined extracts were partitioned in a separatory funnel with water, diethyl ether, and a saturated solution of sodium chloride. The collected ether phase was evaporated to dryness. Each sample of the obtained oleoresin was dissolved in diethyl ether and divided for further analysis. Ten milliliters of extract was saponified with 30% methanolic potassium hydroxide at room temperature in the dark for 24 h. For the removal of soaps and alkalis, the solution was washed with a saturated solution of sodium chloride and distilled water. The organic layer containing carotenoids was dried over anhydrous sodium sulfate and evaporated to dryness. Samples were kept under nitrogen at −20 °C until further use. All experiments were performed under subdued light.

#### HPLC-PDA Analysis of Carotenoids from Apple Peels

**Figure 5:**
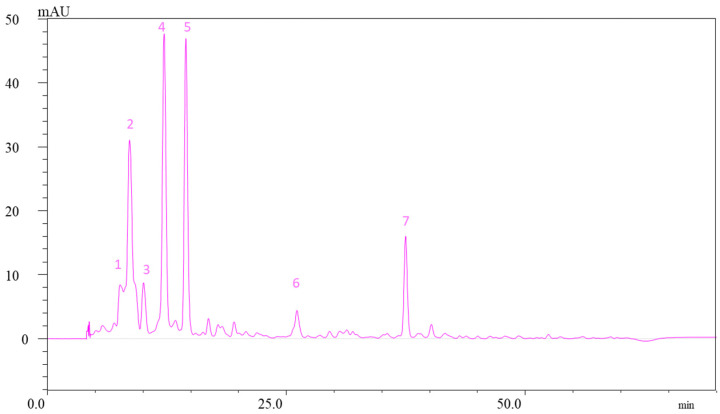
Illustrative chromatogram of carotenoids identified in the peel extract of “Florina”. 1—(all-*E*)-violaxanthin; 2—(all-*E*)-neoxanthin; 3—(all-*E*)-luteoxanthin; 4—(9-*Z*)-violaxanthin; 5—(all-*E*)-lutein; 6—(all-*E*)-β-cryptoxanthin; 7—β-carotene.

The saponified extracts of carotenoids were further diluted with ethyl acetate, filtered through a membrane filter (PTFE, 0.45 µm pore size, Millipore, Germany), and subjected to chromatographic analysis conducted on a Shimadzu high-performance liquid chromatography system equipped with an LC-20 AT binary pump (Prominence), DGU-20 A3 degasser (Prominence), and SPD-M20 photodiode array detector (HPLC-PDA). Carotenoids were separated using a YMC C30 column (5 μm, 24 cm × 4.6 mm) and a mixture of two solvents at a 0.8 mL/min flow rate. Solvent A: methanol/*tert*-butyl methyl ether/water (81:15:4, *v*/*v*/*v*); solvent B: *tert*-butyl methyl ether/methanol/water (90:7:3, *v*/*v*/*v*) (Figure 5). Gradient elution started with 1% B at min 0 and increased to 100% B by min 160 according to the method described by Giuffrida et al. [59]. Carotenoid identification in apple peels was carried out by comparison of the UV–vis spectra, retention time of sample peaks with those of the standards, and literature data. Carotenoid concentration was calculated using the calibration curves of carotenoid standards (calculated in the range of 1–100 μg/mL (lutein R^2^ = 0.991, zeaxanthin R^2^ = 0.996, and β-carotene R^2^ = 0.991).

### 3.3. Determination of Total Phenolics

Total phenolics were measured following the Folin–Ciocalteu colorimetric method from Singleton et al. [60] with some minor modifications. Aliquots (25 µL) of the extracts, gallic acid calibration standard, and water blank were placed into separate plastic cuvettes. Distilled water (1.8 mL) and Folin–Ciocalteu reagent (120 µL) were then added to each cuvette, thoroughly mixed, and incubated for 5 min. After incubation, sodium carbonate (340 µL, 7.5% Na_2_CO_3_ in water) was added, mixed, and allowed to incubate for 90 min at room temperature. Absorption of samples was measured at 750 nm, and TPCs were calculated from the calibration curve, using gallic acid as standard, considering the quantity of peel extracts and dilutions used for the analysis. The calibration curve was generated by preparing gallic acid solutions (0–0.25 mg/mL) and measuring their absorbance at 750 nm. Parameters of the obtained curve: a = 2.364, b = 0.0649, R^2^ = 0.9909. Results were expressed as mg GAE/ kg sample. The analyses were performed in triplicate.

### 3.4. Extraction of Anthocyanins

Apple peels (10 g) were weighed and mixed at 20,000 rpm in a blender (Ultra-Turrax Miccra D-9 KT Digitronic, Bergheim, Germany) with acidified methanol (10 mL, methanol: hydrochloric acid 85:15 [*v*/*v*]). The suspension was centrifuged at 3500 rpm for 10 min. The extract was separated and extracted until the extraction solvent became colorless (total volume of solvent was between 100 and 250 mL). The combined extracts were dried in vacuo at 40 °C. The obtained samples were dissolved in methanol (10 mL), centrifuged at 5000 rpm, and filtered through a membrane filter. The water phase resulting from the extraction was subjected to partitioning using ethyl acetate and then passed through an Amberlite-XAD-7 (1 × 0.5 cm) column previously activated with 6 volumes of water containing 0.3% trifluoroacetic acid (TFA). The column was washed with 3 volumes of water (0.3% TFA) to remove carbohydrates, pectin, and impurities. Anthocyanins and proanthocyanins were eluted with 4 volumes of methanol (0.3% TFA). Anthocyanin fractions were further purified on a Sephadex LH-20 (2.5 × 0.5 cm) column using 10 volumes of a mixture of water/methanol (0.3% TFA) 8:2. Samples containing the pure anthocyanins were dissolved in ultrapure water (5 mL), filtered, and analyzed with the HPLC-PDA system described above.

#### HPLC-PDA Analysis of Anthocyanins

**Figure 6:**
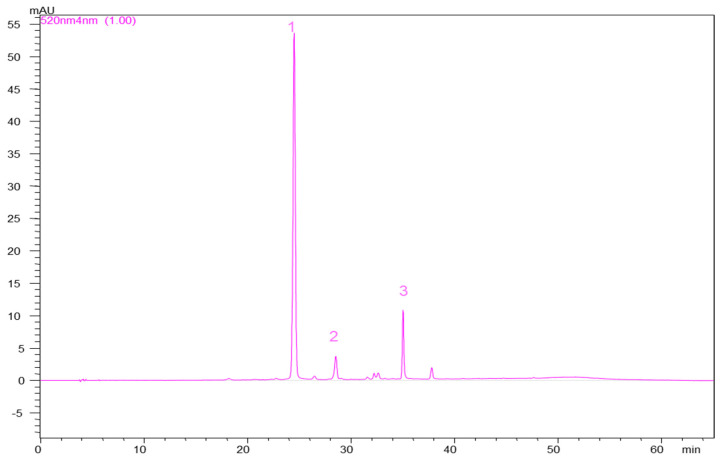
Illustrative chromatogram of anthocyanins identified in the peel extract of “Generos”. 1—cyanidin-3-*O*-galactoside; 2—cyanidin-3-*O*-glucoside; 3—cyanidin-3-*O*-arabinoside.

HPLC analysis was performed on the HPLC-PDA system described above. Separation was achieved on a Luna Phenomenex C18 column (5 µm, 25 cm × 4.6 mm), and column temperature was maintained at 25 °C. The mobile phases were 4.5% formic acid in bidistilled water (solvent A) and acetonitrile (100%) (solvent B), with a solvent flow rate set at 0.5 mL/min. The gradient elution system started with 10% B for 9 min. The percentage of B increased to 12% at 17 min and continued up to 25% B at the 20th min. From the 20th to 55th min, the percentage of B increased until 90%. The absorbance was monitored at 520 nm (Figure 6). Compound identification and peak assignments were achieved based on their retention times, UV–vis spectra, and comparisons to standards and published data. Anthocyanins were quantified using cyanidin-3-*O*-galactoside as standard at concentrations between 2.5 and 500 µg/mL (R^2^ > 0.998).

### 3.5. Determination of Antioxidant Activities

Scavenging effect on ABTS radical: The scavenging ability of all apple peel samples against the radical anion ABTS+ was determined in 96-well plates according to the procedure described [61]. Absorbance of samples was measured at 734 nm after 6 min of incubation in the dark at room temperature, using a microplate reader (BioTek Instruments, Winooski, VT, USA). Results were expressed as µmol TE/g FW.

Ferric reducing antioxidant power (FRAP): Antioxidants are evaluated as reducers of Fe^3+^ to Fe^2+^, which is chelated by 2,4,6-tri(2-pyridyl)s-triazine (TPTZ) to form the Fe^2+^TPTZ complex, with a maximum absorbance at 593 nm [62]. The absorbance of the colored product was monitored using the BioTek Synergy HT spectrophotometer. All solutions were used on the day of preparation. Briefly, TPTZ (2.5 mL, 10 mM in 40 nM HCl), acetate buffer (25 mL, 300 mM, pH = 3.6), and FeCl_3_ (2.5 mL, 20 mM) were mixed. Following the addition of FRAP reagent (180 μL), the mixture was incubated for 3 min. Then, 20 μL of each sample was added to each well, and the absorbance was read immediately at 593 nm with a microplate reader. Samples dilutions were performed when the values were over the linear range of the curve of 0 to 1 μM Fe^2+^/mL, using FeSO_4_ 7H_2_O.

### 3.6. Statistical Analysis

All extractions and chromatographic analyses were performed in triplicate. The results for HPLC, spectrophotometric analyses, and antioxidant assays are presented in tables as the mean ± standard deviation. Significant differences between samples were analyzed with one-way ANOVA post hoc tests, and pairwise multiple comparisons were conducted using Tukey’s test. Significant differences were reported based on *p*  <  0.05. Statistical analyses were performed using the SPSS Statistics 23.0.

Classification of apple cultivars based on their chemical composition (carotenoids, anthocyanins, total polyphenol content, antioxidant activity) was achieved by principal component analysis (PCA) with cross-validation (full model size and center data). In order to give all variables included in the analysis an equal chance to influence the model, we used standardization as the scaling technique. All statistical analyses were performed using Unscrambler X software version 10.5.1 (CAMO Software AS, Oslo, Norway).

## 4. Conclusions

The present study analyzed the phytochemical composition of peel extracts from red- and yellow-skinned apple varieties from Romania. High levels of carotenoid, total phenols, and anthocyanins were cultivar specific and comparable with literature data reported in other studies for the same varieties. Carotenoid concentrations were slightly higher in yellow-skinned varieties compared to red-skinned varieties, though a more pronounced variation occurred at the levels of individual carotenoids. Violaxanthins (all-*E*, 9-*Z*) predominated in red-skinned “Champion” and “Generos” and yellow-skinned “Golden Delicious”, while lutein was the major carotenoid compound in red-skinned “Idared” and yellow-skinned “Reinette Simirenko”, Conversely, total phenolic content was notably higher (ranging between 2056 mg GAE/kg FW (“Generos”) and 2723 mg GAE/kg FW (“Florina”)) in red-skinned varieties, which was partially reflected in the antioxidant capacity of peel extracts, especially in their ABTS radical scavenging potential. “Florina” cultivar with the highest TPC also presented the highest ABTS activity, with yellow-skinned apples having the lowest ABTS potential. All these data further demonstrate the versatility and beneficial effects of fresh apples and encourage their consumption, especially in unpeeled form. Further study may be warranted to better understand the differences in pigments and antioxidant activities among the cultivars during apple storage. These differences may provide useful information for the breeders but also for the consumers.

## Figures and Tables

**Table 1:** Total phenolic and individual anthocyanin contents of red- and yellow-skinned apple varieties.

	Total Phenolic Content (mg GAE/kg FW)	Anthocyanins (mg/kg FW)		
		Cyanidin-3-*O*-galactoside	Cyanidin-3-*O*-glucoside	Cyanidin-3-*O*-arabinoside
Champion	2143 ± 102 ^b^	292 ± 67 ^b^	18.08 ± 9 ^b^	trace
Generos	2056 ± 119 ^c^	175.32 ± 32 ^c^	11.45 ± 6.3 ^c^	23.22 ± 5.4 ^c^
Idared	2209 ± 132 ^b^	289.37 ± 54 ^b^	6.23 ± 0.97 ^d^	40.58 ± 7.2 ^a^
Florina	2723 ± 139 ^a^	396 ± 72 ^a^	29.74 ± 9.21 ^a^	35 ± 6.67 ^b^
Golden Delicious	1600 ± 99 ^d^	nd	nd	nd
Reinette Simirenko	1468 ± 89 ^e^	nd	nd	nd

GAE, gallic acid equivalent; FW, fresh weight; nd, not determined. Different letters within a column denote significant differences (*p* < 0.05).

## Data Availability

The data presented in this study are available on request from the corresponding author.

## References

[1] Boyer J., Liu R.H. (2004). Apple phytochemicals and their health benefits. Nutr. J..

[2] Kalinowska M., Bielawska A., Lewandowska-Siwkiewicz H., Priebe W., Lewandowski W. (2014). Apples: Content of phenolic compounds vs. variety, part of apple and cultivation model, extraction of phenolic compounds, biological properties. Plant Physiol. Biochem..

[3] Wijewardane R.M.N.A., Guleria S.P.S. (2013). Effect of pre-cooling, fruit coating and packaging on postharvest quality of apple. J. Food Sci. Technol..

[4] Duda-Chodak A., Tarko T., Tuszyński T. (2011). Antioxidant activity of apples--an impact of maturity stage and fruit part. Acta Sci. Pol. Technol. Aliment..

[5] Karaman S., Tütem E., Başkan K.S., Apak R. (2013). Comparison of antioxidant capacity and phenolic composition of peel and flesh of some apple varieties. J. Sci. Food Agric..

[6] Shehzadi K., Rubab Q., Asad L., Ishfaq M., Shafique B., Ali Nawaz Ranjha M.M., Mahmood S., Mueen-Ud-Din G., Javaid T., Sabtain B. (2020). A Critical Review on Presence of Polyphenols in Commercial Varieties of Apple Peel, their Extraction and Health Benefits. Op. Acc. J. Bio. Sci. Res..

[7] Tsao R., Yang R., Young J.C., Zhu H. (2003). Polyphenolic profiles in eight apple cultivars using high-performance liquid chromatography (HPLC). J. Agric. Food Chem..

[8] Wolfe K., Wu X., Liu R.H. (2003). Antioxidant activity of apple peels. J. Agric. Food Chem..

[9] Balasuriya N., Rupasinghe H.P. (2012). Antihypertensive properties of flavonoid-rich apple peel extract. Food Chem..

[10] He X., Liu R.H. (2008). Phytochemicals of Apple Peels: Isolation, Structure Elucidation, and Their Antiproliferative and Antioxidant Activities. J. Agric. Food Chem..

[11] Vineetha V.P., Girija S., Soumya R.S., Raghu K.G. (2014). Polyphenol-rich apple (Malus domestica L.) peel extract attenuates arsenic trioxide induced cardiotoxicity in H9c2 cells via its antioxidant activity. Food Funct..

[12] Kheirvari M., Ardekani M.K., Anbara T. (2021). Polyphenol-rich diet, an efficient strategy after bariatric surgery. Obes. Med..

[13] Hyson D.A. (2011). A comprehensive review of apples and apple components and their relationship to human health. Adv. Nutr..

[14] Rasouli H., Farzaei M.H., Khodarahmi R. (2017). Polyphenols and their benefits: A review. Int. J. Food Proper..

[15] Chen Z., Yu L., Liu W., Zhang J., Wang N., Chen X. (2021). Research progress of fruit color development in apple (Malus domestica Borkh.). Plant. Physiol. Biochem..

[16] Lancaster J.E., Dougall D.K. (1992). Regulation of skin color in apples. Crit. Rev. Plant. Sci..

[17] Dar J.A., Wani A.A., Ahmed M., Nazir R., Zargar S.M., Javaid K. (2019). Peel colour in apple (Malus × domestica Borkh.): An economic quality parameter in fruit market. Sci. Hortic..

[18] Peng T., Moriguchi T. (2013). The molecular network regulating the coloration in apple. Sci. Hortic..

[19] Ju Z., Liu C., Yuan Y., Wang Y., Liu G. (1999). Coloration potential, anthocyanin accumulation, and enzyme activity in fruit of commercial apple cultivars and their F1 progeny. Sci. Hortic..

[20] Li X.-J., Hou J.-H., Zhang G.-L., Liu R.-S., Yang Y.-G., Hu Y.-X., Lin J.-X. (2004). Comparison of anthocyanin accumulation and morpho-anatomical features in apple skin during color formation at two habitats. Sci. Hortic..

[21] Merzlyak M.N., Chivkunova O.B. (2000). Light-stress-induced pigment changes and evidence for anthocyanin photoprotection in apples. J. Photochem. Photobiol. B Biol..

[22] Rodriguez-Concepcion M., Avalos J., Bonet M.L., Boronat A., Gomez-Gomez L., Hornero-Mendez D., Limon M.C., Meléndez-Martínez A.J., Olmedilla-Alonso B., Palou A. (2018). A global perspective on carotenoids: Metabolism, biotechnology, and benefits for nutrition and health. Prog. Lipid Res..

[23] Saini R.K., Nile S.H., Park S.W. (2015). Carotenoids from fruits and vegetables: Chemistry, analysis, occurrence, bioavailability and biological activities. Food Res. Int..

[24] Maiani G., Periago Castón M.J., Catasta G., Toti E., Cambrodón I.G., Bysted A., Granado-Lorencio F., Olmedilla-Alonso B., Knuthsen P., Valoti M. (2009). Carotenoids: Actual knowledge on food sources, intakes, stability and bioavailability and their protective role in humans. Mo. Nutr. Food Res..

[25] Molnár P., Deli J., Tanaka T., Kann Y., Tani S., Gyémánt N., Molnár J., Kawase M. (2010). Carotenoids with anti-Helicobacter pylori activity from Golden delicious apple. Phytother. Res..

[26] Toti E., Chen C.Y.O., Palmery M., Villaño Valencia D., Peluso I. (2018). Non-Provitamin A and Provitamin A Carotenoids as Immunomodulators: Recommended Dietary Allowance, Therapeutic Index, or Personalized Nutrition?. Oxidative Med. Cell. Longev..

[27] Roohbakhsh A., Karimi G., Iranshahi M. (2017). Carotenoids in the treatment of diabetes mellitus and its complications: A mechanistic review. Biomed. Pharmacother..

[28] Delgado-Pelayo R., Gallardo-Guerrero L., Hornero-Méndez D. (2014). Chlorophyll and carotenoid pigments in the peel and flesh of commercial apple fruit varieties. Food Res. Int..

[29] Schweiggert R.M., Vargas E., Conrad J., Hempel J., Gras C.C., Ziegler J.U., Mayer A., Jiménez V., Esquivel P., Carle R. (2016). Carotenoids, carotenoid esters, and anthocyanins of yellow-, orange-, and red-peeled cashew apples (*Anacardium occidentale* L.). Food Chem..

[30] Felicetti D.A., Schrader L.E. (2009). Changes in pigment concentrations associated with sunburn browning of five apple cultivars. I. Chlorophylls and carotenoids. Plant Sci..

[31] Muresan E.A., Muste S., Muresan C.C., Mudura E., Paucean A., Stan L., Romina Alina V., Cerbu C.G., Muresan V. (2017). Assessment of polyphenols, chlorophylls, and carotenoids during developmental phases of three apple varieties. Rom. Biotechnol. Lett..

[32] Vondráková Z., Trávníčková A., Malbeck J., Haisel D., Černý R., Cvikrová M. (2020). The effect of storage conditions on the carotenoid and phenolic acid contents of selected apple cultivars. Eur. Food Res. Technol..

[33] Abid M., Jabbar S., Wu T., Hashim M.M., Hu B., Lei S., Zeng X. (2014). Sonication enhances polyphenolic compounds, sugars, carotenoids and mineral elements of apple juice. Ultrason. Sonochem..

[34] Arcos Y., Godoy F., Flores-Ortiz C., Arenas M.A., Stange C. (2020). Boosting carotenoid content in Malus domestica var. Fuji by expressing AtDXR through an Agrobacterium-mediated transformation method. Biotechnol. Bioeng..

[35] Dang Q., Sha H., Nie J., Wang Y., Yuan Y., Jia D. (2021). An apple (Malus domestica) AP2/ERF transcription factor modulates carotenoid accumulation. Hortic. Res..

[36] Edge R., McGarvey D.J., Truscott T.G. (1997). The carotenoids as anti-oxidants—A review. J. Photochem. Photobiol. B Biol..

[37] Wolfe K.L., Liu R.H. (2003). Apple peels as a value-added food ingredient. J. Agric. Food Chem..

[38] Merzlyak M.N., Solovchenko A.E., Smagin A.I., Gitelson A.A. (2005). Apple flavonols during fruit adaptation to solar radiation: Spectral features and technique for non-destructive assessment. J. Plant Physiol..

[39] Merzlyak M.N., Solovchenko A.E., Chivkunova O.B. (2002). Patterns of pigment changes in apple fruits during adaptation to high sunlight and sunscald development. Plant Physiol. Biochem..

[40] Solovchenko A.E., Avertcheva O.V., Merzlyak M.N. (2006). Elevated sunlight promotes ripening-associated pigment changes in apple fruit. Postharvest Biol. Technol..

[41] Solovchenko A.E., Chivkunova O.B., Merzlyak M.N., Gudkovsky V.A. (2005). Relationships between chlorophyll and carotenoid pigments during on- and off-tree ripening of apple fruit as revealed non-destructively with reflectance spectroscopy. Postharvest Biol. Technol..

[42] Jakopic J., Stampar F., Veberic R. (2009). The influence of exposure to light on the phenolic content of ‘Fuji’ apple. Sci. Hortic..

[43] Nagy A., Riczu P., Tamás J. (2016). Spectral evaluation of apple fruit ripening and pigment content alteration. Sci. Hortic..

[44] Wang X., Li C., Liang D., Zou Y., Li P., Ma F. (2015). Phenolic compounds and antioxidant activity in red-fleshed apples. J. Funct. Foods.

[45] Duda-Chodak A.D.A., Tarko T., Satora P., Sroka P., Tuszynski T. (2010). The profile of polyphenols and antioxidant properties of selected apple cultivars grown in Poland. J. Fruit Ornam. Plant Res..

[46] Loncaric A., Matanovic K., Ferrer P., Kovac T., Sarkanj B., Skendrovic Babojelic M., Lores M. (2020). Peel of Traditional Apple Varieties as a Great Source of Bioactive Compounds: Extraction by Micro-Matrix Solid-Phase Dispersion. Foods.

[47] Chinnici F., Bendini A., Gaiani A., Riponi C. (2004). Radical scavenging activities of peels and pulps from cv. Golden Delicious apples as related to their phenolic composition. J. Agric. Food Chem..

[48] Massini L., Rico D., Martin-Diana A.B., Barry-Ryan C. (2013). Valorisation of Apple Peels. Eur. Food Res. Rev..

[49] Assumpção C.F., Hermes V.S., Pagno C., Castagna A., Mannucci A., Sgherri C., Pinzino C., Ranieri A., Flôres S.H., Rios A.d.O. (2018). Phenolic enrichment in apple skin following post-harvest fruit UV-B treatment. Postharvest Biol. Technol..

[50] Bars-Cortina D., Macià A., Iglesias I., Romero M.P., Motilva M.J. (2017). Phytochemical Profiles of New Red-Fleshed Apple Varieties Compared with Traditional and New White-Fleshed Varieties. J. Agric. Food Chem..

[51] Kokalj D., Zlatić E., Cigić B., Kobav M.B., Vidrih R. (2019). Postharvest flavonol and anthocyanin accumulation in three apple cultivars in response to blue-light-emitting diode light. Sci. Hortic..

[52] Sadilova E., Stintzing F.C., Carle R. (2006). Chemical quality parameters and anthocyanin pattern of red-fleshed Weirouge apples. J. Appl. Bot. Food Qual..

[53] Awad M.A., de Jager A. (2002). Formation of flavonoids, especially anthocyanin and chlorogenic acid in ‘Jonagold’ apple skin: Influences of growth regulators and fruit maturity. Sci. Hortic..

[54] Wijewardane R.M.N.A., Guleria S.P.S. (2009). Combined Effects of Pre-cooling, Application of Natural Extracts and Packaging on the Storage Quality of Apple (Malus domestica) cv. Royal Delicious. Trop. Agric. Res..

[55] Kunradi-Vieira F.G., Da Silva Campelo Borges G., Copetti C., Da Valdemiro Gonzaga L., Costa Nunes E., Fett R. (2009). Activity and contents of polyphenolic antioxidants in the whole fruit, flesh and peel of three apple cultivars. Arch. Latinoam. Nutr..

[56] Vinson J.A., Su X., Zubik L., Bose P. (2001). Phenol antioxidant quantity and quality in foods: Fruits. J. Agric. Food Chem..

[57] Bahukhandi A., Dhyani P., Bhatt I.D., Rawal R.S. (2018). Variation in Polyphenolics and Antioxidant Activity of Traditional Apple Cultivars from West Himalaya, Uttarakhand. Hortic. Plant. J..

[58] Schlatterer J., Breithaupt D.E. (2006). Xanthophylls in commercial egg yolks: Quantification and identification by HPLC and LC-(APCI)MS using a C30 phase. J. Agric. Food Chem..

[59] Giuffrida D., Pintea A., Dugo P., Torre G., Pop R.M., Mondello L. (2012). Determination of Carotenoids and their Esters in Fruits of Sea Buckthorn (*Hippophae rhamnoides* L.) by HPLC-DAD-APCI-MS. Phytochem. Anal..

[60] Singleton V.L., Orthofer R., Lamuela-Raventós R.M. (1999). Analysis of total phenols and other oxidation substrates and antioxidants by means of folin-ciocalteu reagent. Meth Enzymol.

[61] Arnao M.B., Cano A., Alcolea J.F., Acosta M. (2001). Estimation of free radical-quenching activity of leaf pigment extracts. Phytochem. Anal..

[62] Benzie I.F., Strain J.J. (1996). The ferric reducing ability of plasma (FRAP) as a measure of "antioxidant power": The FRAP assay. Anal. Biochem..

